# Colocynth and Colocynthian Reflections

**Published:** 1877-11

**Authors:** C. L. Gish

**Affiliations:** Shopiere, Wis.


### "COLOCYNTH AND COLOCYNTHIAN REFLECTIONS."

SHOPIERE, Wis., October, 1877.

In a very learned article, with the above heading, Dr. Tucker, in your last issue, gives us the result of his reflections, which is in his mind, doubtless, an original discovery, to wit: "That colocynth will allay the pain caused by excessive peristaltic action, better than any drug in use, not excepting opium, providing it be used in the proper dose."

Now if Dr. Tucker will take up "Bæhr's Therapeutics," (homeopathic) and turn to page 473, he will find these words: "The treatment of such a colic is very satisfactory. We possess a remedy against it which is almost always effective, whereas every other method of treatment seems without any avail. We mean *Colocynth.*" And by turning to the Materia Medica of Hemple, Hering, Burt, and other Homeopathic writers, he will find that colocynth has held a place high in their estimation in this class of disorders, including diarrhœa and dysentery.

Thus we often find that what at first seems to us to be an original discovery, ultimately proves to be but a "bequest from antiquity." And the Doctor will, upon investigation, find that the hydragogue action of colocynth has not been the limit of its usefulness. C. L. Gish, M. D.

